# Enhancement of α-Mangostin Wound Healing Ability by Complexation with 2-Hydroxypropyl-β-Cyclodextrin in Hydrogel Formulation

**DOI:** 10.3390/ph13100290

**Published:** 2020-10-02

**Authors:** Nasrul Wathoni, Diah Permata Sari, Ine Suharyani, Keiichi Motoyama, Ahmed Fouad Abdelwahab Mohammed, Arief Cahyanto, Marline Abdassah, Muchtaridi Muchtaridi

**Affiliations:** 1 Department Pharmaceutics and Pharmaceutical Technology, Faculty of Pharmacy, Universitas Padjadjaran, Sumedang 45363, Indonesia; de.permatasr@gmail.com (D.P.S.); ine18001@mail.unpad.ac.id (I.S.); marline.abdassah@unpad.ac.id (M.A.); 2 Department of Pharmaceutics, School of Pharmacy Muhammadiyah Cirebon, Cirebon 45153, Indonesia; 3 Graduate School of Pharmaceutical Sciences, Kumamoto University, Kumamoto 8620973, Japan; motoyama@kumamoto-u.ac.jp; 4 Department of Pharmaceutics, Faculty of Pharmacy, Minia University, Minia 61519, Egypt; Ahmed.mohamed1@minia.edu.eg; 5 Department of Dental Materials Science and Technology, Faculty of Dentistry, Universitas Padjadjaran, Sumedang 45363, Indonesia; arief.cahyanto@fkg.unpad.ac.id; 6 Department of Pharmaceutical Analysis and Medicinal Chemistry, Faculty of Pharmacy, Universitas Padjadjaran, Sumedang 45363, Indonesia

**Keywords:** α-Mangostin, hydroxypropyl-β-cyclodextrin, inclusion complex, hydrogel, wound healing

## Abstract

α-Mangostin (α-M), one of the active compounds in *Garcinia mangostana* peel, has been effectively used in wound healing. However, its poor solubility in aqueous solution causes low bioavailability for skin ulcers, hindering its application in wound healing. The aim of this study was to improve the solubility of α-M through complex formation with 2-hydroxypropyl-β-cyclodextrin (α-M/HP-β-CD CX) and to evaluate the healing activity of the complex. The α-M/HP-β-CD CX was incorporated in a sodium carboxymethylcellulose hydrogel (α-M/HP-β-CD CX HG), and the in vivo healing activity was examined in mice. Evaluation of α-M/HP-β-CD CX HG, including organoleptic evaluation, homogeneity, pH, spreadability, swelling ratio, consistency, scanning electron microscopy (SEM), and in vitro drug release, was carried out. The complex formation of α-M/HP-β-CD CX was confirmed by FTIR and PXRD analysis. The solubility of the α-M/HP-β-CD CX in water linearly increased about 11.7-fold compared to α-M alone, and by 3.5-fold compared to the α-M/HP-β-CD physical mixture (α-M/HP-β-CD CX PM). The α-M/HP-β-CD CX HG was homogenous, the pH was found to be in the neutral range, the spread area was 5 cm, and the consistency was stable until 14 days. SEM analysis showed that α-M/HP-β-CD CX HG surged due to the porous structure of the HG. In addition, in vitro release of α-M from α-M/HP-β-CD CX HG was considerably increased compared to α-M/HP-β-CD PM HG and α-M HG. Notably, in vivo evaluation in mice showed that α-M/HP-β-CD CX HG significantly accelerated the wound healing ability compared to other HGs. Thus, α-M/HP-β-CD CX HG has potential as a new formulation of α-M for wound healing therapy.

## 1. Introduction

Several herbal plants have been developed for wound healing because of their safety and remarkable pharmacological activity [1]. One of the plants which have been used in wound healing is *Garcinia mangostana* peel, an Indonesian native plant containing more than 50 xanthone compounds. This plant contains α-Mangostin, which has been used for a long time in wound care [2,3,4].

Wounds are a skin disruption caused by chemical, microbial, and physical trauma, as well as fire, etc. The wound healing process occurs in four phases, i.e., the inflammatory and hemostatic, migration, proliferation, and remodeling phases [5]. α-M, one of the xanthone compounds, has become of considerable interest in wound treatment due to its anti-inflammatory effect [6,7].

However, the poor water solubility of α-M hinders its application in several preparations. Various methods to increase the solubility have been developed, such as micronization, chemical modification, pH adjustment, and complexation [8]. Hydroxypropyl-β-cyclodextrin (HP-β-CD) has the ability to form an inclusion complex due to its unique structure. HP-β-CD has a truncated conical structure with a hydrophobic cavity capable of entrapping the hydrophobic molecule and a hydrophilic outside. In addition, HP-β-CD is a hydroxyalkyl derivative, which has better solubility than other cyclodextrins [9]. Previous studies showed that the solubility of alpinetin, curcumin, and rutin in aqueous solutions was increased using the inclusion complex with HP-β-CD [10,11,12].

Wound healing therapies are classified into traditional healing agents, i.e., topical pharmaceutical formulations (liquid and semi-solid), traditional dressings (gauze and bandage), and modern dressings (hydrocolloid, alginates, and hydrogel (HG)). Gauze is a woven or non-woven fabric which can be combined with drugs in a suitable carrier, but limits microbial growth and leaves a second trauma when the gauze is removed from the ulcer. Foam provides a high surface area for water absorption, resulting in an insufficient wound treatment [13]. The liquid form has a short residence time on the wound, particularly in suppurated wounds. Moreover, ointments are ineffective, especially in exuded wounds [5]. Modern wound dressings are the outcome of traditional healing developments in swelling properties, residence time, and drug delivery [5].

HG is one type of modern wound dressing that can be used in wound healing applications due to its biodegradability, biocompatibility, and favorable permeability. This form has the ability to minimize the irritation surrounding the tissue because of the smooth and moist surface of the HG [14,15]. Sodium carboxymethylcellulose (Na-CMC), a cellulose polymer, has been used in wound healing due to its hydrophilic, non-allergenic, non-toxic, and biocompatible qualities [16]. In addition, HP-β-CD is widely used as a linker in HG preparations underlying Diels–Alder reactions in water, resulting in the improvement of HG properties [17].

Recently, in vitro and in vivo studies of the α-Mangostin (α-M) and HP-β-CD complex have not been observed, especially in HG formulation for wound healing applications. In this study, the first solubility improvement of α-M was investigated by complex formation with HP-β-CD. The complex was incorporated into Na-CMC HG to increase residence time and bioavailability in the wound healing process.

## 2. Results

### 2.1. Characterization of α-M/HP-β-CD CX

#### 2.1.1. Fourier Transform Infrared Spectroscopy (FTIR)

**Figure 1:**
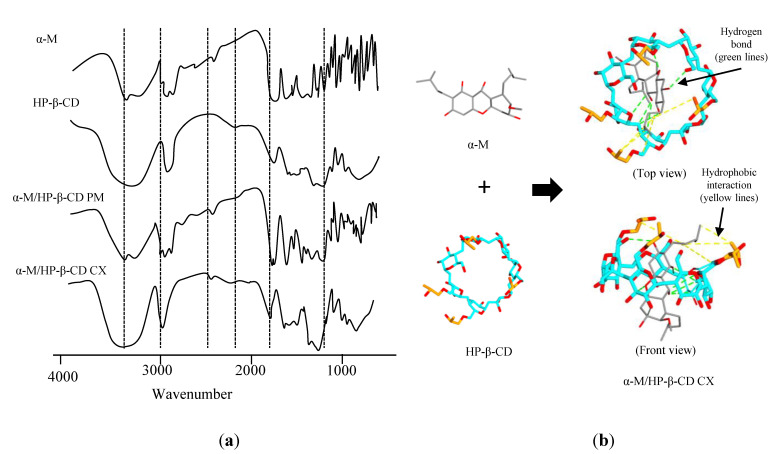
FTIR study of α-M/HP-β-CD CX (**a**), and the molecular docking of α-M/HP-β-CD CX (**b**).

FTIR analysis was performed to determine the functional group and the interaction that probably occurred in α-M/HP-β-CD CX. The result of FTIR evaluation (Figure 1a) showed the various intensities, shifts, and peaks of α-M/HP-β-CD CX spectra in the fingerprint region 1450–500 cm^−1^. The α-M spectra indicated the absorption band at 1033.83 cm^−1^ (C–O–C stretching vibration), 1373.32 cm^−1^ (C–O stretching vibration), and 2380.87 cm^−1^ (C–H stretching vibration). The HP-β-CD spectra indicated absorption at 1643.35 cm^−1^ (H–O–H stretching vibration), 2075.41 cm^−1^ (C–H bending), 2927.94 cm^−1^ (C-H stretching vibration), and 3394.72 cm^−1^ (O–H stretching vibration) [18].

To predict the interaction between α-M and HP-β-CD, molecular docking was performed. Figure 1b illustrates that α-M can interact with several functional groups of HP-β-CD (binding affinity −6.0 kcal/mol), e.g., –CO−, −CH2, and OH. There are five hydrogen bondings between the alkyl group in α-M and the alkyl group from hydroxypropyl substituents in HP-β-CD. In addition, hydrophobic interactions occurred between the alkyl group in α-M and the alkyl group from hydroxypropyl substituents in HP-β-CD.

#### 2.1.2. Powder X-ray Diffraction (PXRD)

**Figure 2:**
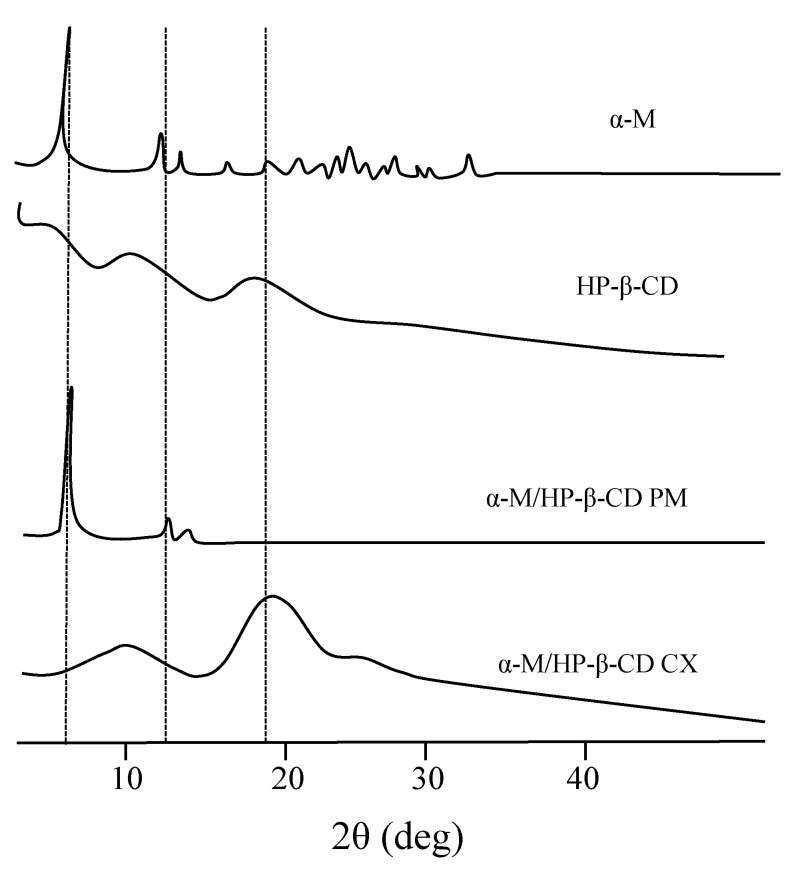
PXRD study of α-M/HP-β-CD CX.

The PXRD patterns of α-M, HP-β-CD, α-M/HP-β-CD PM, and α-M/HP-β-CD CX can be seen in Figure 2. The intense crystalline peaks of α-M are shown below 40°. Two of the characteristic crystalline peaks of α-M can still be detected in α-M/HP-β-CD PM at 5° and 14°, while α-M/HP-β-CD CX did not show any of the crystalline peaks.

#### 2.1.3. Solubility of α-M/HP-β-CD CX

**Figure 3:**
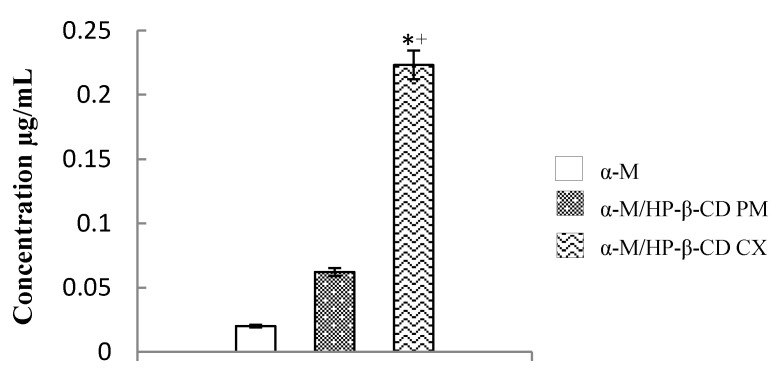
Solubility study of α-M/HP-β-CD CX. * *p* < 0.05, compared to the α-M. + *p* < 0.05, compared to the α-M/HP-β-CD PM.

The solubility was studied by dissolving the samples in deionized water and then filtering. The degree of solubility (Figure 3) of α-M/HP-β-CD CX improved linearly about 11.7 fold compared to α-M, and 3.5 fold compared to α-M/HP-β-CD PM, while the solubility of α-M and α-M/HP-β-CD PM was 0.019 µg/mL.

### 2.2. Preparation and Characterization of α-M/HP-β-CD CX HG

**Figure 4:**
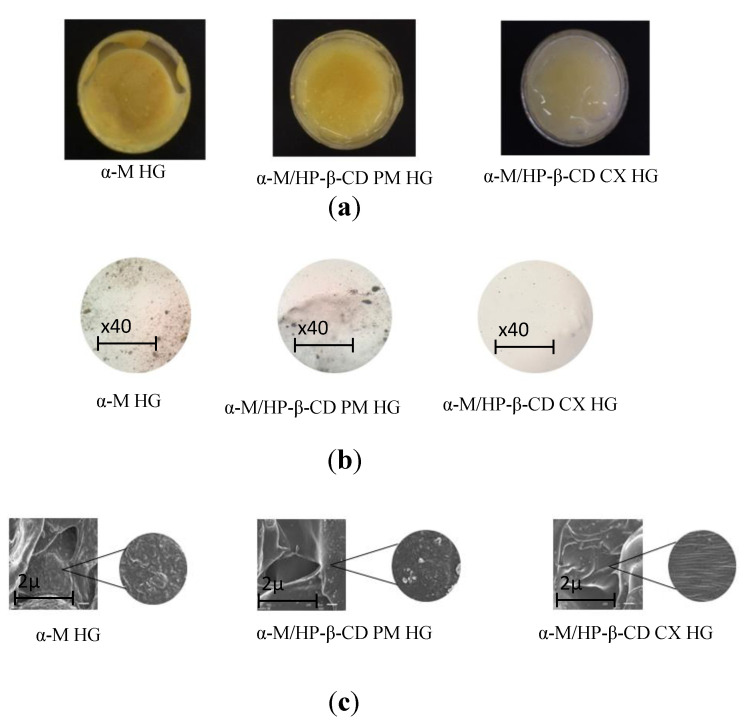
Characterization of α-M/HP-β-CD CX HG: organoleptic (**a**), homogeneity, magnification of ×40 (**b**), morphology, magnification of ×300 (**c**).

α-M/HP-β-CD CX HG was successfully fabricated with Na-CMC as a gel base. An organoleptic evaluation was performed to determine the visual appearance of the preparation. Figure 4a shows that the HG containing α-M/HP-β-CD CX has a transparent yellowish color, compared to α-M HG and α-M/HP-β-CD PM HG, which remain insoluble materials [19].

The homogeneity of the preparations was observed using a microscope to observe whether or not the HG preparation showed a homogeneous appearance with no grains (Figure 4b). The α-M/HP-β-CD CX HG showed a homogenous gel appearance compared to α-M HG and α-M/HP-β-CD PM HG, which showed an inhomogeneous mass.

SEM analysis was performed to determine the morphological properties of the HGs. The sample was coated with gold-palladium for 10 s during the analysis. The morphologies of α-M HG, α-M/HP-β-CD PM HG, and α-M/HP-β-CD CX HG can be seen in Figure 4c.

**Figure 5:**
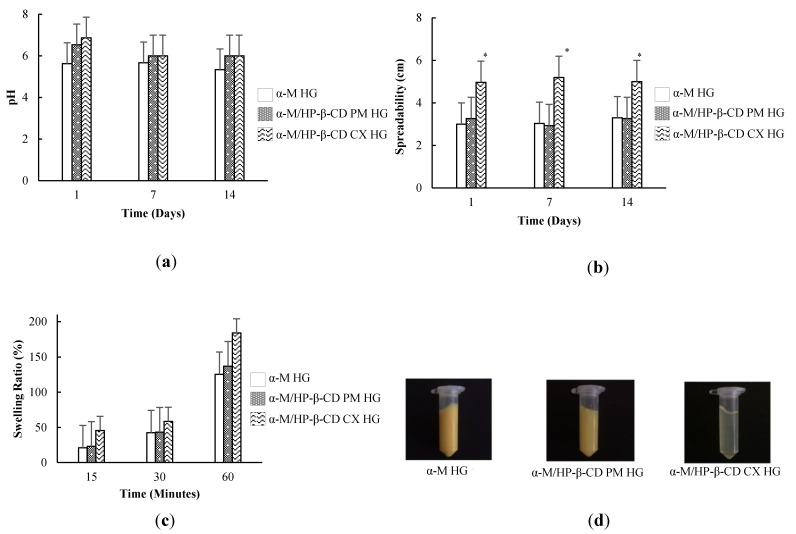
Characterization of α-M/HP-β-CD CX HG: (**a**) pH, (**b**) spreadability test, (**c**) swelling ratio, (**d**) consistency. (*n* = 3). * *p* < 0.05, compared to the α-M HG.

The HGs should have a pH in the acceptable pH skin range to avoid irritation on the skin (Figure 5a). The pH of α-M HG on the 1st, 7th, and 14th day was 5.63 ± 1.08, 5.67 ± 1.58, and 5.33 ± 1.58, respectively.

The spreadability of the HGs at 1, 7, and 14 days can be seen in Figure 5b. Diameter of the spreading area of α-M/HP-β-CD CX HG was quite high compared to other HGs, reaching about 5.20 ± 0.14, 5.00 ± 0.14, and 5.08 ± 0.21 cm at 1, 7, and 14 days, respectively.

The swelling ratio of α-M/HP-β-CD CX HG was the greatest compared to the other HGs (Figure 5c). The swelling ratio of α-M HG, α-M/HP-β-CD PM HG, and α-M/HP-β-CD CX HG at 15 and 30 minutes were below 100%, but exceeded 100% after 60 min.

The consistency of HGs was evaluated to determine their physical properties. Consistency evaluation was done through the centrifugal method (Figure 5d). The results showed that all the preparations did not separate.

### 2.3. In Vitro Drug Release

**Figure 6:**
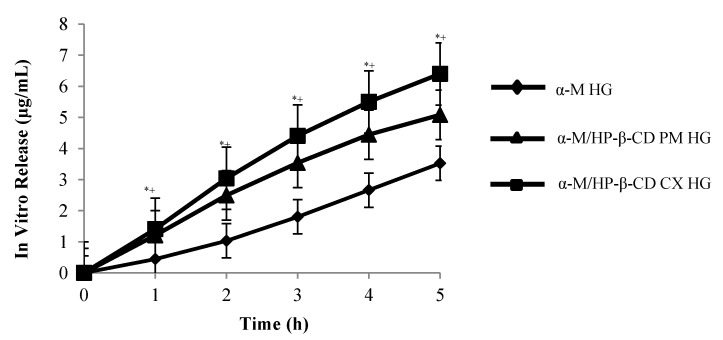
In vitro release of α-M from α-M/HP-β-CD CX HG in PBS pH 7.4. (*n* = 3) * *p* < 0.05, compared to α-M, + *p* < 0.05 and α-M/HP-β-CD PM.

The results showed that α-M/HP-β-CD CX HG significantly increased the release of α-M from α-M/HP-β-CD CX HG compared to other HGs (Figure 6).

The drug release from α-M/HP-β-CD CX HG was followed by a biphasic curve which showed the first release at the 1st hour (1.048 µg/mL), and until the 5th hour (6.397 µg/mL), with the regression parameters showing that the correlation coefficient (r) was 0.99 (Table 1).

### 2.4. In Vivo Wound Healing Activity

**Figure 7:**
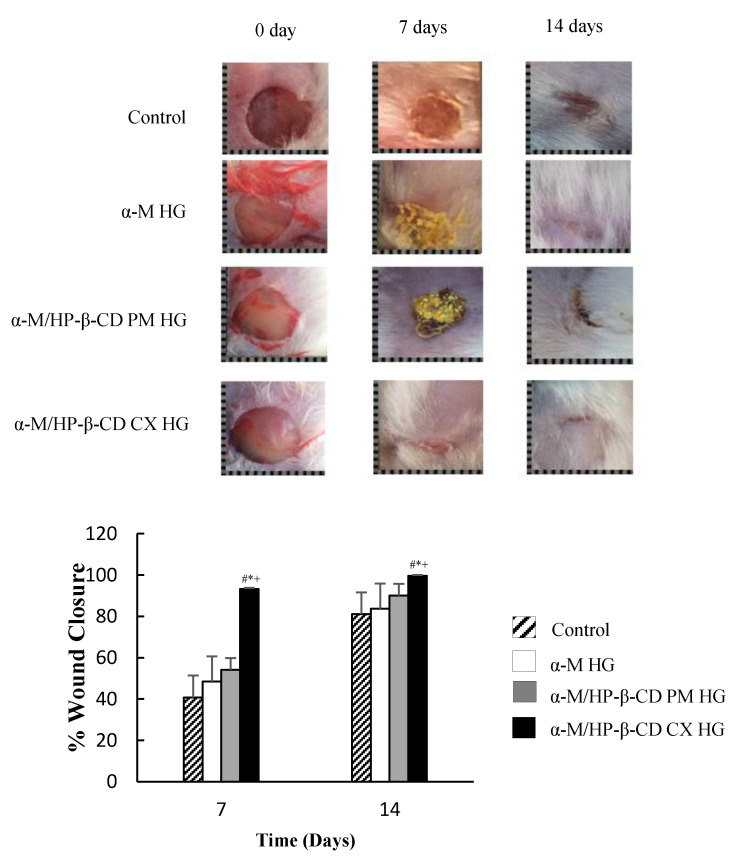
Wound closure study of α-M/HP-β-CD CX HG (*n* = 3) * *p* < 0.05 compared to the control, # *p*< 0.05 compared to α-M HG, and + *p* < 0.05 compared to α-M/HP-β-CD PM HG.

Finally, to clarify the efficacy of α-M/HP-β-CD CX HG on wound healing ability, the in vivo study was performed on mice. Mice were divided into four treatment groups (Figure 7). The wound closure was calculated using Equation (2). The α-M/HP-β-CD CX HG wound closure at 7 and 14 days showed the significantly accelerated wound healing ability of α-M in α-M/HP-β-CD CX HG compared to other groups (control, α-M HG, and α-M/HP-β-CD PM HG groups).

## 3. Discussion

### 3.1. Characterization of α-M/HP-β-CD CX

#### 3.1.1. Fourier Transform Infrared Spectroscopy (FTIR)

We successfully prepared α-M/HP-β-CD CX as a fine light-yellow powder. FTIR analysis was performed to determine the functional group and the interaction that probably occurred in α-M/HP-β-CD CX. The absorption at >3000 nm showed a sloping spectrum. The spectra of α-M/HP-β-CD CX showed that the fingerprint of α-M at the wavelengths 2924.09 cm^−1^ and 3431.72 cm^−1^ disappeared, indicating a physical or chemical interaction occurred due to the complexation of α-M and HP-β-CD. In addition, molecular docking predicted the interactions between α-M and HP-β-CD, including five hydrogen bonds and one hydrophobic interaction in α-M/HP-β-CD CX.

The complex formation between HP-β-CD CX with various drugs depends on the ability of drug molecules to enter the HP-β-CD cavity. The inclusion process did not break the covalent bonds, but some molecular interactions might have occurred during the complex formation, i.e., hydrophobic interaction, van der Waals interaction, hydrogen bond, and the release of high energy water from the HP-β-CD cavity [20,21,22]. The complex formation began when the drug molecule and HP-β-CD approached each other and initiated with a fission of water from the HP-β-CD cavity. Furthermore, the interaction between the hydroxyl group (–OH) in the ring of α-M and the carbonyl group (C–O) formed a hydrogen bond. Reconstruction of water molecules will form around drug molecules not covered by HP-β-CD [20].

#### 3.1.2. Powder X-ray Diffraction (PXRD)

PXRD studies were done to identify the crystallinity of the α-M in complex α-M/HP-β-CD CX. The PXRD patterns of α-M, HP-β-CD, α-M/HP-β-CD PM, and α-M/HP-β-CD CX showed the successful complex formation between α-M and HP-β-CD CX. An amorphous pattern was seen in HP-β-CD and α-M/HP-β-CD CX. The diffractogram of α-M/HP-β-CD CX was more amorphous than α-M/HP-β-CD PM. Figure 2 shows that the intense peak below 40° is the crystalline characteristic of α-M. The diffraction pattern of α-M/HP-β-CD PM displayed the same peak with α-M/ at 5° and 14°, indicating that the crystallinity was still detectable. This intense peak disappeared in α-M/HP-β-CD CX, indicating that α-M was probably entrapped in the cavity of HP-β-CD [17,23]. The spectra indicated that the interaction between α-M and HP-β-CD probably formed a number of hydrogen bonds and changed the physical characteristic of the drug in the complex [24]. In a previous study, the cavity of HP-β-CD was able to entrap the α-M molecule due to its hydrophobicity. In this study, it is suggested that the complex was formed through a weak hydrogen bond between the hydroxyl group (–O6) in ring A of α-M and the hydroxy group (O6) in HP-β-CD at the narrow cavity [25,26].

#### 3.1.3. Solubility of α-M/HP-β-CD CX

The solubility of α-M/HP-β-CD CX significantly improved compared to α-M and α-M/HP-β-CD PM. The hydrophilic surface surrounding the outer HP-β-CD molecules was able to improve the solubility of the drug (α-M) entrapped within the cavity of HP-β-CD. Even though the increasing of solubility was achieved in α-M/HP-β-CD PM, there was no hydrogen bond formed between α-M and HP-β-CD PM. This suggested that the interaction between both molecules was due to the interaction between the hydroxypropyl group of the HP-β-CD molecule with α-M [25]. Related research has also stated that the solubility of epothilone increased to about 0.082 µg/mL in HP-β-CD complex [27]. These results corresponded to Loftsson, 2005, who stated that Higuchi and Connors classified the complex based on its solubility improvement in aqueous solution [28]. However, further studies confirmed that the effect of complexation on the molar absorptivity of α-M would be necessary as complexation is known to affect the photophysical properties of encapsulated guests in aqueous solution.

### 3.2. Preparation and Characterization of α-M/HP-β-CD CX HG

α-M/HP-β-CD CX HG was incorporated in Na-CMC hydrogel, resulting in a yellowish gel. Na-CMC is a “smart” cellulose due to its sensitivity to various ionic strength and pH. The presence of sodium in the CMC structure provides electrostatic charges as a linker in HG formation and creates a double effect swelling ratio. Na-CMC plays an important role in the repulsion between the similar electric charge of long polymer chains that form a neutral network, leading to the enhancement of swelling capacity. In addition, the presence of counterions provides an increasing number of water molecules entering the network due to the Donnan effect [16]. 

The homogeneity evaluation was done by applying the HG on a glass slide, covering it with another one, and then observing it under a microscope. The HG containing α-M/HP-β-CD CX had a more homogenous appearance compared to α-M/HP-β-CD PM, which showed the grains. Homogeneity was also affected by the water solubility of the active substance to be dispersed in the HG [29]. In addition, the syneresis phenomenon of α-M/HP-β-CD CX HG did not occur.

The results of SEM strongly elaborated on previous results showing the homogeneity of α-M/HP-β-CD CX HG. The α-M particles appeared to disappear in α-M/HP-β-CD CX HG, suggesting that α-M/HP-β-CD CX was successfully prepared and dissolved within the Na-CMC gel base.

The pH of the HGs was observed to confirm the safety of the preparation when the HG was applied to the skin. The pH of α-M HG was found to be relatively stable during storage. On the other hand, the pH of α-M/HP-β-CD PM HG and α-M/HP-β-CD CX HG decreased at 7 days. The factors affecting the pH are environment, temperature, and storage [30]. The presence of water in HG allows α-M ionization to liberate a hydrogen ion, underlying the pH decrease of the HG. Although the pH of the preparations decreased on the 7th day, all the preparation were stable until 14 days in the skin pH range (4.5–6.5) [30,31].

Spreadability was investigated to determine the ability of the HGs to spread on the skin. When HGs spread and cover skin ulcers, proliferation will occur. The preparation containing α-M/HP-β-CD CX had the highest spreadability due to the solubility effect from the active substance. In an aqueous solution, Na-CMC released Na^+^ and replaced it with H^+^, and a greater cohesion force caused the lower spread area of α-M HG and α-M/HP-β-CD PM HG [32].

The swelling ratio of the HGs revealed the swelling ability of the HGs to entrap water molecules in their polymer network. The presence of HP-β-CD in α-M/HP-β-CD PM HG and α-M/HP-β-CD CX HG increased the swelling ratio of the HG due to the ability of HP-β-CD to bind the water molecules. Swelling ratio is related to the ability of the HG to absorb the exudate of the skin ulcer, thus speeding up the healing process. Swelling ability accelerates the wound healing process by maintaining a moist environment around the wound [33].

The separated materials at the bottom of the tube were insoluble material from α-M HG and α-M/HP-β-CD PM HG. The consistency caused by the concentration of Na-CMC in the range of 1–10% was able to maintain the consistency of the HG preparation [34]. Interestingly, the α-M was successfully dissolved in α-M/HP-β-CD CX HG. These results confirmed previous solubility studies of α-M/HP-β-CD CX. However, there was no significant difference in the statistic as physicochemical characterization was observed in the HG formulation, suggesting Na-CMC as the base of hydrogel plays a crucial role in each formulation.

### 3.3. In Vitro Drug Release

To determine the release of drug from the HGs, the in vitro release study of α-M from α-M/HP-β-CD CX HG was inspected. The drug release from α-M/HP-β-CD CX HG followed a biphasic curve, which showed the first release at the 1st hour (1.048 µg/mL) and until the 5th hour (6.397 µg/mL), with the regression parameters showing that the correlation coefficient was (r) 0.99 (Table 1).

The drug release followed Fickian diffusion, which illustrated that the drug release was not dependent on drug concentration [34]. These results suggest that the higher the release of α-M/HP-β-CD CX HG, the higher the bioavailability of α-M when applied to the skin. In addition, HP-β-CD has the ability to improve the permeation of active substances into the skin [35,36].

### 3.4. In Vivo Wound Healing Activity

The wound healing activity of α-M/HP-β-CD CX HG was almost 100% in 7 days, and the wound was completely closed in 14 days, while the α-M HG treated group still showed an open wound. The soluble complex in water caused faster drug release to the wound, resulting in a faster healing process than the α-M and α-M/HP-β-CD PM without the complex. The healing process occurred without the solubilization of the drug at the ulcer, but was directly distributed onto the ulcer to give the healing activity.

The previous study stated that synthetic compounds from α-M derivatives, i.e., 3-*O*-methyl mangostin, 3,6-di-*O*-methyl mangostin, mangostin triacetate, 1-isomangostin, mangostin-3,6-di-*O*-(tetraacethyl)-glucoside, and mangostin-3,6-di-*O*-glucoside, significantly reduced inflammation in rats [37]. In addition, α-M showed a faster inhibition and treatment of skin ulcers. The initial phase was associated with histamine and serotonin release. The second phase released prostaglandin, where it was proposed that α-M was capable of inhibiting PGE_2_ release [38]. Moreover, the antioxidant activity of the α-M could assist the proliferation process in wound healing stages [39]. The presence of β- and HP-β-CD increased both the skin permeability and stability of α-mangostin entrapped in the inside of the cyclodextrin cavity, resulting in the protection effect from the skin metabolism [9]. Thus, the cyclodextrin acted as the penetrant enhancer to deliver the mangostin molecules at the skin wound. In a previous study, HG was applied to the wound site to deliver the curcumin/HP-γ-CyD complex. The curcumin/HP-γ-CyD complex HG significantly increased the wound healing ability of curcumin [40]. In this study, α-M/HP-β-CD CX HG with the Na-CMC as a superabsorbent polymer showed an adequate wound healing ability.

Considering the properties of the HGs in wound healing therapy, permeable HGs allow the exchange of CO_2_, O_2_, and H_2_O, in which the HGs have the characteristics of 90% water and 10% polymer, with a high-water content. Thus, they are well fitted to treating dry and necrotic wounds. Moist environment in the HG could improve the process and facilitate the resolution of injury debridement effectively through the HG’s absorption ability [39].

## 4. Materials and Methods

### 4.1. Material

α-M was procured from Chengdu Biopurify Phytochemicals Ltd. (Chengdu, China). HP-β-CD (DS 4) was obtained from Nihon Shokuhin Kako (Tokyo, Japan). Na-CMC, glycerin, propylene glycol, and methylparaben were bought from Brataco (Jakarta, Indonesia). All the reagents were of analytical grade and used without further purification.

### 4.2. Methods

#### 4.2.1. Preparation of α-M/HP-β-CD CX

The inclusion complex of α-M/HP-β-CD (α-M/HP-β-CD CX) was prepared by α-M and HP-β-CD in a ratio of 1:1 as follows: HP-β-CD powder (1205 g) was dissolved in 20 mL of deionized water. In a separate flask, α-M (0.410 g) was dissolved in 30 mL of methanol. The α-M solution was slowly added into the HP-β-CD solution, which was mixed and sonicated for 15 min, then mixed at 120 rpm for 48 h at room temperature. The solvent was evaporated using a rotary evaporator (65 rpm, 70 °C). Furthermore, the concentrated solution was dried in the oven at 50 °C for 12 h [41]. The physical mixture of α-M/HP-β-CD (α-M/HP-β-CD PM) was prepared by a simple method in which both powders were mixed and ground for 5 min [17].

#### 4.2.2. Evaluation of α-M/HP-β-CD CX

##### Fourier Transform Infrared Spectroscopy (FTIR) and Molecular Docking

The functional groups in α-M, HP-β-CD, α-M/HP-β-CD PM, and α-M/HP-β-CD CX were determined by FTIR (Shimadzu’s IRPrestige-21) analysis in accordance with the following procedures: each sample of α-M, 2-HP-β-CD, α-M/HP-β-CD PM, and α-M/HP-β-CD CX was crushed with KBr and compressed to form a pellet. Each pellet containing the sample was then scanned in the range of 400–4000 cm^−1^ [18].

The 3D structure of HP-β-CD (DS 4) was established through the 3D structure modeling process using a Chem3D Pro 12.0 from β-CD (PDB ID: 4RER) [42]. The structure of 3D α-M was obtained from PubChem [43]. All structures were minimized using Molecular Mechanical 2 (MM2) minimization [42]. The docking process was carried out using the PyRx [44] and AutoDock Vina [45]. In the settings, HP-β-CD was used as a macromolecule, and α-M was used as a ligand. α-M and HP-β-CD were rigid during the molecular docking assay. The processing result was then stored as a macromolecular complex. Results were visualized using the BIOVIA Discovery Studio^®^ 2020 Client system [46]. The interaction between the ligand and its macromolecule was determined using the ligand–receptor interaction setting.

##### Powder X-ray Diffractometry (PXRD)

PXRD (PANalytical X’Pert Pro) analysis was performed to determine the crystallinity of α-M, HP-β-CD, α-M/HP-β-CD PM, and α-M/HP-β-CD CX. Briefly, each sample (5 mg) of α-M, α-M/HP-β-CD PM, and α-M/HP-β-CD CX was placed on a sample holder, and the tests were conducted using the diffractometer with filtered Cu-K_α_ radiation (40.0 kV, 40.0 mA), scanning rate 2°/minute at 2θ, and a diffraction angle of 3° to 50° [47].

##### Solubility Study

Each sample (60 mg) of α-M, α-M/HP-β-CD PM, and α-M/HP-β-CD CX was dissolved in 25 mL of deionized water (Milli-Q), and stirred at 120 rpm at a temperature of 25 °C for 48 h. The mixtures were centrifuged (Hettich EBA 200) at 3500× *g* for 30 min, and filtered with a syringe filter at 0.45 µm. The concentration of α-M was determined using UV spectrophotometry at 246 nm [18].

#### 4.2.3. Preparation of Na-CMC HG

The HG was composed of Na-CMC as a base, and the excipients included glycerin, propylene glycol, and methylparaben (Table 2). The HG was prepared with the following procedures: Na-CMC was poured into warm water (70 °C) and mixed until homogeny. Each of the α-M formulations (25 mg of α-M, α-M/HP-β-CD CX, α-M/HP-β-CD PM) were added into the base and mixed until homogeny. Methylparaben, glycerin, and propylene glycol were added gradually. The mixture was stirred until a homogeneous solution was formed.

#### 4.2.4. Evaluation of α-M/HP-β-CD CX HG

##### Organoleptic Test

Organoleptic evaluation was performed by human sense organs to study the visual appearance of the preparation. Samples (20 g) of α-M HG, α-M/HP-β-CD CX HG, and α-M/HP-β-CD PM HG were placed in a 25 mL glass beaker at room temperature and were visually analyzed by a human panel, consisting of 3 trained postgraduate students from the Faculty of Pharmacy, Universitas Padjadjaran [48,49].

##### pH Evaluation

The pH evaluation was carried out using a pH meter (Mettler Toledo S220). Briefly, 1% of each sample (α-M HG, α-M/HP-β-CD CX HG, and α-M/HP-β-CD PM HG) was diluted with 20 mL of distilled water in a 25 mL glass beaker, and then the pH was measured using a pH meter. The pH should have been in the range of 4.5–6.5 [30,31].

##### Homogeneity Test

To clarify the formulation process of α-M HG, α-M/HP-β-CD CX HG, and α-M/HP-β-CD PM HG preparations, the homogeneity test was performed using a microscope (Olympus CX23). A few samples were dispersed over a microscope glass slide and closed by a glass cover. Each sample was observed under the microscope (magnification of ×40). The samples were taken from 3 parts of the preparation, i.e., the upper, middle, and bottom of the preparation [29,50].

##### Spreadability

Samples (0.5 g) of α-M HG, α-M/HP-β-CD CX HG, and α-M/HP-β-CD PM HG were placed in the center of a Petri dish in an upside-down position to form a circle of 2 cm in diameter, which was signed as an initial diameter. The other Petri dish was employed in reverse position. A weight of 150 g was added above the upper dish for 5 min, and the final diameter was measured. The diameter of the spreadability area should have been in the range of 5–7 cm [31,48,51].

##### Scanning Electron Microscopy (SEM)

The morphological structure of the HG was studied by SEM (JEOL JSM 6510 LA). Briefly, 2 g of each sample (α-M HG, α-M/HP-β-CD CX HG, and α-M/HP-β-CD PM HG) was lyophilized and then freeze-dried. The samples were attached to a sample holder and coated with gold-palladium for 10 s, then observed by SEM (magnification of ×300) [17,52,53].

##### Swelling Ratio

Samples (0.5 g) of (α-M HG, α-M/HP-β-CD CX HG, and α-M/HP-β-CD PM HG) were immersed in phosphate buffer saline (PBS) solution pH 7.4 for 15, 30, and 60 min at room temperature. The swelling ratio was determined by the following Equation (1) [54,55].

(1) $$\mathrm{Swelling}\;\mathrm{Ratio}=\frac{\left(W_{s}-W_{d}\right)}{W_{d}}\times 100\%$$

where *W*_d_ is the weight of the first HG, and *W*_s_ is the weight of the swollen HG at a certain time.

##### Consistency

To study the consistency alteration of the HGs, the consistency test was carried out using a centrifuge (Eppendorf Centrifuge 5424 R). The HG was inserted into the centrifuge tube to as much as 2 mL. The α-M HG, α-M/HP-β-CD CX HG, and α-M/HP-β-CD PM HG were centrifuged at 1800× *g* for 5 h [56].

##### In Vitro Drug Release

To investigate the drug release mechanism, the in vitro release study was done by dissolving 0.2 g of samples (α-M HG, α-M/HP-β-CD CX HG, and α-M/HP-β-CD PM HG) in 20 mL PBS pH 7.4 and mixed. Then, 5 mL of the release medium was taken at various time points and replaced with the same volume of fresh PBS. The α-M amount released from each sample was measured using UV spectrophotometry at 246 nm [57].

##### In Vivo Wound Healing Activity

The wound healing activity of the α-M HG, α-M/HP-β-CD CX HG, and α-M/HP-β-CD PM HG was examined in mice. The procedures were approved by the Ethical Committee of Medical Faculty Universitas Padjadjaran, with ethical approval number 1036/UN6.KEP/EC/2018. The anesthesia (2.5 mg/kg BW of Valisanbe intramuscularly) was injected before the evaluation to minimize pain in the animal tests [58].

In vivo healing activity was performed in Swiss–Webster strain mice (*Mus musculus*) that were 2–3 months old and weighed 20–30 g. The number of mice used was 20, divided into four groups, with 5 mice in each group. The animals were placed at a constant temperature (26 ± 1 °C) before the evaluation [58]. The hair of the mice was shaved, then their back skin was swabbed with 70% alcohol, and two full-thickness wounds were created on the back of each mouse using a sterile biopsy punch (8 mm in diameter) [40].

Each sample of HG was applied to the wound and evaluated every day. The surface of the wound was covered with a sterile gauze. To evaluate the wound closure of the skin tissue, the measurement of the wound area in the experimental animals was carried out at a 7 and 14-day interval. The level of wound closure was calculated using the formula Equation (2) below [58].

(2) $$\%\;\mathrm{of}\;\mathrm{wound}\;\mathrm{closure}=\frac{\mathrm{initial}\;\mathrm{wound}\;\mathrm{area}-\mathrm{wound}\;\mathrm{area}\;\mathrm{at}\;t\;\mathrm{day}}{\mathrm{initial}\;\mathrm{wound}\;\mathrm{area}}\;\times \;100\%$$

## 5. Conclusions

In this study, α-M/HP-β-CD CX HG was successfully prepared and confirmed by FTIR, PXRD, and solubility studies. α-M/HP-β-CD CX was an amorphous complex which significantly improved solubility by about 11.7 fold compared to α-M alone, and by 3.5 fold compared to α-M/HP-β-CD PM. The better solubility of α-M/HP-β-CD CX created improved properties of α-M/HP-β-CD CX HG compared to α-M HG and α-M/HP-β-CD PM HG. The pH was found to be in the neutral range, the spread area was 5 cm, and the consistency was stable until 14 days. In addition, in vitro and in vivo studies of α-M/HP-β-CD CX HG showed the linear correlation in enhancement of α-M wound healing ability by complexation with HP-β-CD in Na-CMC HG formulation. These results suggest that α-M/HP-β-CD CX HG has potential as a new formulation of α-M for wound healing therapy.

## Figures and Tables

**Table 1:** Regression parameters of Higuchi from the percentage of drug release and quadratic curve time of α-M release from α-M HG, α-M/HP-β-CD PM HG, and α-M/HP-β-CD HG CX in PBS (pH 7.4) in 5 h.

Parameter	α-M HG	α-M/HP-β-CD PM HG	α-M/HP-β-CD CX HG
Slope (% min-0.5)	1.25 ± 0.13	1.55 ± 0.06	2.02 ± 0.01
Correlation Coefficient (r)	0.96 ± 0.002	0.99 ± 0.0002	0.99 ± 0.00

**Table 2:** Preparation of hydrogel (HG).

Formulation	α-M HG	α-M/HP-β-CD PM HG	α-M/HP-β-CD CX HG
α-M	25 mg	-	-
α-M/HP-β-CD PM	-	25 mg	-
α-M/HP-β-CD CX	-	-	25 mg
Na-CMC	0.4 g	0.4 g	0.4 g
Glycerin	2 g	2 g	2 g
Propylene glycol	1 g	1 g	1 g
Methylparaben	0.03 g	0.03 g	0.03 g
Distilled water added	20 mL	20 mL	20 mL
